# Severe type 1 upgrading leprosy reaction in a renal transplant recipient: a paradoxical manifestation associated with deficiency of antigen-specific regulatory T-cells?

**DOI:** 10.1186/s12879-017-2406-9

**Published:** 2017-04-24

**Authors:** Ana Paula Vieira, Maria Angela Bianconcini Trindade, Flávio Jota de Paula, Neusa Yurico Sakai-Valente, Alberto José da Silva Duarte, Francine Brambate Carvalhinho Lemos, Gil Benard

**Affiliations:** 1 Laboratory of Medical Investigation Unit 56, Division of Clinical Dermatology, Medical School, University of São Paulo, São Paulo, Brazil; 2 Health Institute, São Paulo State Health Department, São Paulo, Brazil; 3 Renal Transplantation Service, Clinics Hospital, Medical School, University of São Paulo, São Paulo, Brazil; 4 Laboratory of Medical Investigation Unit 53, Tropical Medicine Institute, University of São Paulo, São Paulo, Brazil

**Keywords:** Leprosy, Type 1 reaction, Renal transplantation, Immunosuppressors, Immune reconstitution syndrome, Regulatory T cell

## Abstract

**Background:**

Due to its chronic subclinical course and large spectrum of manifestations, leprosy often represents a diagnostic challenge. Even with proper anti-mycobacteria treatment, leprosy follow up remains challenging: almost half of leprosy patients may develop reaction episodes. Leprosy is an infrequent complication of solid organ transplant recipients. This case report illustrates the challenges in diagnosing and managing leprosy and its reactional states in a transplant recipient.

**Case presentation:**

A 53-year-old man presented 34 months after a successful renal transplantation a borderline-tuberculoid leprosy with signs of mild type 1 upgrading reaction (T1R). Cutaneous manifestations were atypical, and diagnosis was only made when granulomatous neuritis was found in a cutaneous biopsy. He was successfully treated with the WHO recommended multidrug therapy (MDT: rifampicin, dapsone and clofazimine). However he developed a severe T1R immediately after completion of the MDT but no signs of allograft rejection. T1R results from flare-ups of the host T-helper-1 cell-mediated immune response against *Mycobacterium leprae* antigens in patients with immunologically unstable, borderline forms of leprosy and has been considered an inflammatory syndrome in many aspects similar to the immune reconstitution inflammatory syndromes (IRS). The T1R was successfully treated by increasing the prednisone dose without modifying the other immunosuppressive drugs used for preventing allograft rejection. Immunological study revealed that the patient had a profound depletion of both in situ and circulating regulatory T-cells and lack of expansion of the Tregs upon *M. leprae* stimulation compared to T1R leprosy patients without iatrogenic immunosuppression.

**Conclusions:**

Our case report highlights that leprosy, especially in the transplant setting, requires a high degree of clinical suspicion and the contribution of histopathology. It also suggests that the development of upgrading inflammatory syndromes such as T1R can occur despite the sustained immunosuppressors regimen for preventing graft rejection. Our hypothesis is that the well-known deleterious effects of these immunosuppressors on pathogen-induced regulatory T-cells contributed to the immunedysregulation and development T1R.

**Electronic supplementary material:**

The online version of this article (doi:10.1186/s12879-017-2406-9) contains supplementary material, which is available to authorized users.

## Background

Due to its chronic and subclinical progression, leprosy often represents a diagnostic challenge and depends on a high degree of suspicion by the clinician [1]. Even with proper anti-mycobacteria treatment, leprosy follow up remains challenging. Almost half of leprosy patients develop reaction episodes, i.e., worsening of the previous lesions or appearance of new inflamed lesions and neuritis due to exacerbation of the immune response in the patient. The difficulties in diagnosing and managing leprosy are particularly evident in the transplant setting, where leprosy is not initially suspected and the diagnosis is only revealed through histopathology examination of cutaneous lesions [2].

Post-transplant leprosy is an infrequently reported infectious complication in SOT recipients [2]. However, its frequency in this population may increase for several reasons. First, despite WHO initiatives to reduce leprosy transmission, it remains highly endemic in certain countries and can even increase due to relaxation of leprosy surveillance services and other socio-political issues [3]. Second, several highly endemic countries (e.g., Brazil and India) have been witnessing an increase in their transplant recipient population [4]. In Brazil, for instance, more than 5000 kidney transplants are performed yearly [5] Third, leprosy in transplant patients may also become an issue in developed countries due to increased population movements from underdeveloped to developed countries (and vice versa). Finally, increased survival and more aggressive immunosuppressive therapy may also increase the rate of infectious conditions such as leprosy that are caused by relatively low virulence pathogens with long periods of incubation [6].

This case report illustrates the challenges in diagnosing and managing leprosy and its reactional states in a transplant recipient. In addition, we were able to follow up some aspects of regulatory T-cells (Tregs) in peripheral blood from the patient and in situ, which may give some insight into the paradox of an individual who develops an exacerbation of an antigen-specific immune response while under sustained and potent immunosuppression.

## Case presentation

**Fig. 1:**
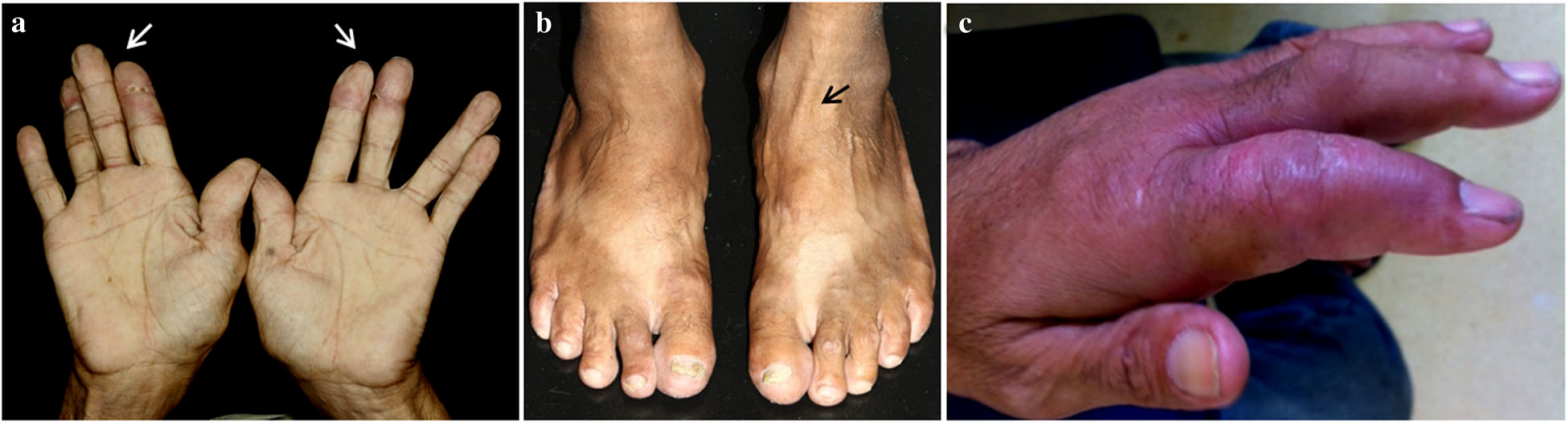
Cutaneous lesions at the time of diagnosis of leprosy (**a** and **b**) and during the severe type 1 reaction (**c**). **a** Erythematous papules on the middle and proximal phalanges of fingers on both hands, some of them ulcerated and with crusts (*arrows*). **b** Tibial nerve enlargement (*arrow*). **c** Erythemato-violaceous papules and severe edema of the hand

**Fig. 2:**
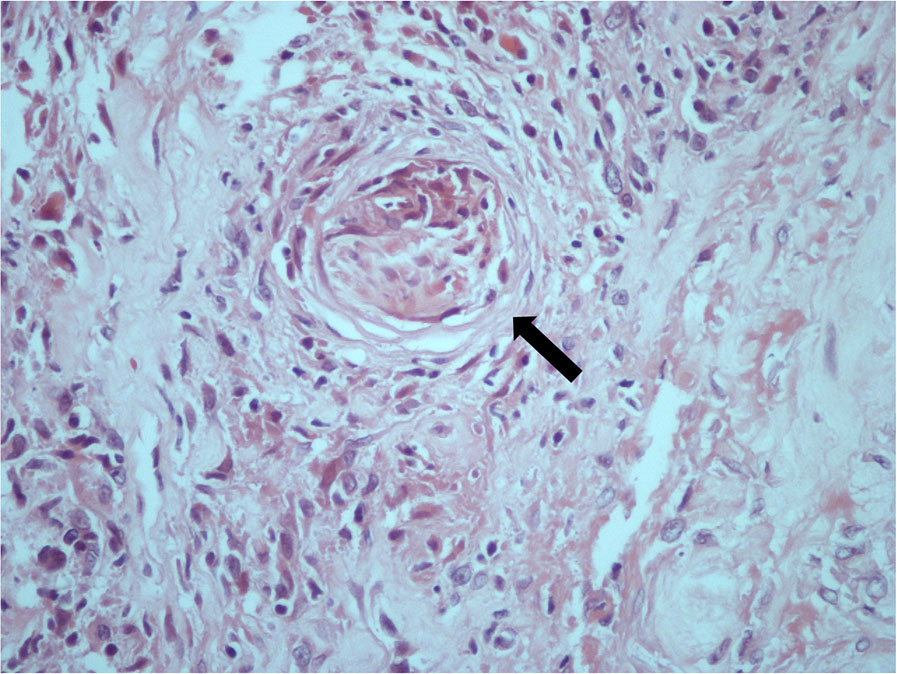
Cutaneous biopsy taken for diagnosis of leprosy showing an inflammatory infiltrate in the dermis with a granuloma within a nerve (*arrow*) (400×)

A 53-year-old man with end-stage renal disease due to hypertensive nephrosclerosis received a kidney from his sister in 2009. No induction therapy was used, and immunosuppressive therapy (IT) maintenance consisted of prednisone (5 mg/day), mycophenolate sodium (1080 mg/day) and tacrolimus trough levels of 5–7 ng/mL. Thirty-four months post-transplant he developed erythematous papules on the middle and proximal phalanges of fingers on both hands. Some of the papules were ulcerated and with crusts (Fig. 1a). A biopsy of a skin lesion showed chronic epithelioid granulomas with neuritis on the dermis, a lack of bacilli by Fite-Faraco staining but a positive immunohistochemistry staining (IHC) for BCG on the nerves (Fig. 2). A diagnosis of BT leprosy was made. The characteristics of the skin lesions, resembling vasculitis, and the presence of giant cells and mild edema permeating the granulomas in the biopsy were suggestive of a mild type 1 upgrading reaction [7]. A subsequent re-evaluation disclosed left ulnar and tibial nerve enlargement and loss of sensation in the affected areas of the hands (Fig. 1b).

He denied contact with individuals with known leprosy, and examination of close relatives was negative for leprosy, including the sister who donated the kidney graft; she is doing well and has never developed signs or symptoms suggestive of leprosy. Two biopsies were taken from the kidney graft during the patient’s follow up; histopathology analysis of both did not disclose alterations compatible with leprosy. The patient was born in the countryside of Bahia State, an area of leprosy high endemicity, but moved to São Paulo city in 1977. São Paulo state was classified as a high endemicity area up to ~1996 but since then there was a marked reduction in the number of cases and it has been reclassified as a low endemicity area.

The patient started 12 months of multidrug therapy (MDT: rifampicin 300 mg/month, dapsone 100 mg/day and clofazimine 100 mg/day). During all MDT, the immunosuppression remained unchanged, except for the dosage of prednisone, which was kept at 10 mg/day instead of 5 mg/day. The patient completed the MDT treatment, with full resolution of the skin lesions and partial improvement of hand anesthesia. One month after completion of MDT, he presented with painful erythemato-violaceous papules and edema of both hands, especially of the fingers of the left hand, with marked functional impairment (Fig. 1c). A biopsy of a left hand lesion disclosed a chronic granulomatous dermatitis characterized by intense lymphomonocytic infiltrate, epithelioid granulomas, edema and focal areas of fibrous exudation, features typical of upgrading T1R leprosy. With this diagnosis, prednisone was increased to 40 mg/day. Mycophenolate sodium and tacrolimus were maintained, but one month later, mycophenolate was removed due to persistent diarrhea. Four weeks later, as the manifestations of the upgrading T1R were partially improved, the prednisone dose was tapered, and everolimus was introduced to replace mycophenolate sodium. By week 16, the prednisone dose was reduced to 5 mg/day, and the upgrading T1R achieved complete remission. The patient remains without relapses at the time of this publication. However, because of the development of proteinuria, everolimus was subsequently removed, and the patient remained on prednisone 5 mg/day and tacrolimus, with trough levels between 5 and 8 ng/mL. Renal function was stable throughout follow up (serum creatinine ≤1.5 mg/dL; estimated glomerular filtration rate ≥ 52 mL/min/1.73 m^2^).

### Tregs evaluation

Circulating Treg numbers were measured by flow cytometry and in situ (biopsies) by IHC (Table 1) (see Additional file 1 with the strategy and methods for the Treg measurements). Written informed consent was obtained from the patient before performing the immunoassays and the study was approved by the Ethics Committee of the Clinics Hospital (protocol #107.460). The patient’s results were compared with those recently reported in a group of BT leprosy patients with severe upgrading T1R but not under immunosuppression (T1R group, *n* = 14) [8]. The in situ frequency of Tregs in the first skin lesion biopsy obtained for diagnosis of leprosy while the patient was undergoing a mild reaction (71 cells/mm^2^) further decreased when he developed a severe reaction (55 cells/mm^2^). Both frequencies were much lower than the results observed in the biopsies taken during a severe reaction episode in the T1R group (170 ± 23 cells/mm^2^).

**Table 1:** Frequency of Tregs in situ (lesions biopsy), ex vivo (peripheral blood), and in in vitro stimulated PBMC, and CTLA-4 expression by the Tregs, in the transplant recipient as compared with the T1R group

	In situ (biopsy) cells/mm^2^	Ex vivo (PB) %		In vitro (expansion) %					
	Treg	Treg^a^	CTLA-4^b^	Treg^a^			CTLA-4^b^		
				Medium	*ML*	PHA	Medium	*ML*	PHA
At diagnosis	71	-	-	-	-	-	-	-	-
Severe reaction	55	0.8	4.1	0.03	0.93	3.0	0.0	0.3	33.6
Remission (4 months)	-	1.2	6.0	1.0	1.72	15.5	0.2	0.9	44.5
Remission (2 years)	-	2.35	-	2.0	2.65	22.8	-	-	-
T1R group^c^	170 ± 23	3.4 ± 0.4	24.9 ± 4.8	2.3 ± 0.3	6.9 ± 0.7	18.9 ± 1.4	13.8 ± 3.6	17.8 ± 4.7	72 ± 5.9

*PB* peripheral blood, *PBMC* peripheral blood mononuclear cells, *Treg* regulatory T-cells, *ML Mycobacterium leprae* antigen, *PHA* phytohemaglutinnin, *T1R* type 1 upgrading leprosy reaction

^a^Percentage of FoxP3^+^CD127^low/−^ among CD4^+^CD25^+^ cells

^b^Percentage of CTLA-4^+^ cells among Tregs

^c^Patients with severe T1R without immunosuppressors (*n* = 14). Results presented as mean ± SEM

Peripheral blood Tregs could be assessed only during severe T1R and in two moments after its remission. The patient had very low levels of circulating Tregs (CD4^+^CD25^+^CD127^low/−^Foxp3^+^ cells) both during the severe reaction (0.76%) and after its remission (1.2%) 4 months later, compared with the 3.4 ± 0.4% of circulating Tregs in the T1R group. Over two years after the reaction episode, while at a higher tacrolimus trough level (7 ng/mL), the percentage of Tregs persisted at a subnormal level (2.35%).

We also examined the capacity of the circulating Tregs to expand in response to a non-specific stimulus (phytohemagglutinin) and to *M. leprae*. In agreement with the ex vivo and in situ low Treg numbers, during the reaction episode, the Tregs of the patient exhibited markedly reduced expansion with both stimuli (3.0 and 0.93%), compared to the T1R group (18.9 ± 1.4 and 6.9 ± 0.7%). Of note, the expansion driven by *M. leprae* remained hindered at each of the three times it was tested, while the expansion driven by phytohemagglutinin increased to “normal” levels after remission of the severe reaction.

A functional study of the Tregs of the patient was not possible due to insufficient cell yield. However, we were able to measure the Tregs expression of CTLA-4, a molecule tightly related to their suppressive capacity [9]. Both during reaction and after remission, few Tregs expressed this molecule either ex vivo or in vitro compared to the T1R group.

## Discussion

Leprosy can represent a difficult diagnosis because of its chronic subclinical course and large spectrum of manifestations. Since these manifestations are determined at least partly by the immune response of the patient, one would expect atypical leprosy presentations in transplant recipients; however, most cases reported to date in SOT recipients presented “regular” manifestations of the infection [2, 10]. Conversely, the patient described here developed skin lesions resembling vasculitis that did not raise the suspicion of leprosy. The diagnosis of BT leprosy occurred due to the biopsy of a cutaneous lesion. This “atypical” presentation was probably related to the mild upgrading T1R presented by the patient. Signs of nerve involvement suggestive of leprosy, such as anesthesia and nerve enlargements, were only detected in a subsequent dermatologic reevaluation. In fact, cutaneous biopsy frequently plays a decisive role in the diagnosis of leprosy. However, in T1R leprosy, the cutaneous lesions may harbor no or an insufficient number of bacilli to be revealed even by appropriate (e.g., Fite-Faraco) staining. Pathologists should therefore rely on the presence of neuritis, which is not always evident because the inflammatory response can result in the destruction of nerves. In such instances, leprosy can be easily misdiagnosed as sarcoidosis or other granulomatous inflammatory reactions.

Surprisingly, the patient already presented signs of a mild T1R at diagnosis, which subsided with MDT alone and became severe just after completion of the MDT. T1R results from flare-ups of the Th-1 cell-mediated immune response of the host against *M. leprae* antigens in patients with immunologically instable, borderline forms of leprosy [11]. This diagnosis is observed during MDT but it is also diagnosed either before or after MDT [11]. Paradoxical inflammatory exacerbation in leprosy patients has been described following BCG vaccination, probably as a result of increased *M. leprae*-reactive cell mediated immunity due to the boosting of the cell-mediated immunity by homologues *M. leprae* antigens present in BCG [12]. Interestingly, in many of these patients it was associated with T1R. Similarly, “paradoxical” T1R was also reported in paucibacillary patients who have been treated with dapsone alone: the T1R that these patients developed after finishing the treatment was ascribed to withdrawal of the drug, since dapsone has been shown to exhibit immunosuppressive activity [13]. Hence, these paradoxical reactions can be regarded as inflammatory syndromes in many aspects similar to the IRS syndromes [14, 15]. The term IRS was originally used to describe the pathogen-associated inflammatory syndrome presented by AIDS patients undergoing immune reconstitution secondary to highly active antiretroviral therapy. However, IRS-like syndromes have also been described in non-HIV patients, including patients with chronic granulomatous diseases other than leprosy such as tuberculosis and paracoccidioidomycosis, who experienced immune-mediated, paradoxical clinical deterioration during their follow up [16–18]. In addition, IRS-like syndromes have been described in post-transplant recipients during antimicrobial treatment of specific infectious conditions [19]. The few cases of transplant recipients with leprosy reactions reported in the literature represented challenging diagnosis and management [2, 10, 14, 20]. The development of a reaction was in general linked to tapering of immunosuppression, although this was not true for every case [10, 21] and the present case report.

The mechanisms underlying the immune response flare ups in T1R leprosy patients remain to be investigated. In AIDS patients, the following two mutually non-exclusive mechanisms can trigger IRIS: exacerbation of the pathogen-induced T-cell mediated effector response and failure of its regulation by antigen-specific Tregs [22]. In the post-transplant setting, IRS has been linked to reversal of the anti-inflammatory to the pro-inflammatory responses as a result of withdrawal or tapering of the IS regimen [14, 19]. In the present case report, the severe upgrading T1R was not due to modifications in the immunosuppressive regimen used for preventing graft rejection, although cessation of the leprosy MDT with dapsone could have contributed in part to it [23]. Our hypothesis is that the severe upgrading T1R was related mainly to the deficient regulation of the *M. leprae* antigen-specific effector responses by Tregs.

The different classes of immunosuppressors target different T-cell activation pathways, but share the blockade of T-cell-mediated effector responses as the main mechanism to prevent allograft rejection. However, Tregs have also been shown to play an important role in graft survival; deficient recruitment of Tregs to the allograft was associated with poor graft outcome [24]. The immunosuppressors have markedly different effects on Tregs. Calcineurin inhibitors block the expansion/function of Tregs, while mTor agents such as everolimus do not, favoring expansion and functional preservation of these cells [25]. The effect of mycophenolate on Tregs is less clear, but recent data show that at therapeutic concentrations, it potently blocks allospecific Treg function/expansion [26]. In agreement with this, our patient showed strikingly reduced in situ and circulating Tregs during the severe reaction episode compared to a group of leprosy patients with severe T1R but not under any immunosuppression. The expansion of Tregs in vitro was also blocked, particularly when induced with *M. leprae*. With T1R resolution, Tregs reacquired a “normal” rate of expansion upon non-specific stimulation, but with *M. leprae,* it remained severely curtailed. The patient was immunosuppressed with tacrolimus and mycophenolate throughout leprosy treatment. Unfortunately, Tregs were not assayed in the short period that everolimus replaced mycophenolate, although it has been shown that everolimus fails to abrogate the inhibitory effect of tacrolimus on Tregs [27]. Moreover, although we could not perform functional assays of the Tregs, we found that the Tregs of the patient had extremely reduced expression of CTLA-4, a molecule that has been associated with Treg suppressive function in transplant recipients [9]. Overall, these data suggest that immunosuppressors, besides acting on effector responses, can also block the induction of pathogen-specific Tregs and result in IRS in transplant recipients by deregulating the balance between the effector and regulatory responses. Interestingly, the absence of the signs of graft rejection in this patient suggests that the balance between allospecific effectors vs. regulatory responses and between pathogen-induced effectors vs. regulatory responses would be differentially regulated.

The rationale for the treatment of IRS or other IRS-like syndromes is control of the inflammatory process. Non-steroidal anti-inflammatory drugs can be used, and in severe cases, corticosteroids are indicated [16–19]. Similarly, severe T1R requires prompt corticosteroid therapy as it can lead to nerve destruction, being the leading cause of permanent sequels in leprosy. Our patient was successfully treated by increasing the prednisone dose without modifying the other two immunosuppressive drugs. He resolved the T1R without sequels except for persistent mild anesthesia in the affected areas of the hands.

## Conclusion

In summary, the present case report highlights the challenge of diagnosing and managing leprosy in a kidney transplant recipient. Diagnosis of leprosy, especially in the transplant setting, requires a high degree of suspicion and the contribution of histopathology. The patient developed severe T1R despite the sustained immunosuppressive regimen for preventing graft rejection. Our hypothesis is that the deficiency of pathogen-induced Tregs due to the deleterious effects of the immunosuppressors contributed to this inflammatory syndrome. Further studies are warranted to better understand the role of Tregs in IRS, as they can potentially help to improve the diagnosis and management of this still challenging syndrome.

## Additional file

Additional file 1: Strategy and methods for the Treg measurements. (DOC 1009 kb)

## Abbreviations

BCG: Bacillus Calmette-Guérin
BT: Borderline tuberculoid
CTLA-4: Cytotoxic T-lymphocyte-associated protein 4
IHC: Immunohistochemistry
IRS: Immune reconstitution syndrome
IT: Immunosuppressive therapy
MDT: Multidrug therapy
MMF: Mycophenolate mofetil
mTOR: Mammalian target of rapamycin
PB: Peripheral blood
PHA: Phytohemagglutinin
SOT: Solid organ transplant
T1R: Type 1 reaction
Tregs: Regulatory T-cells
WHO: World Health Organization
