# Supplementary material for: Severe type 1 upgrading leprosy reaction in a renal transplant recipient: a paradoxical manifestation associated with deficiency of antigen-specific regulatory T-cells?
Source: BMC Infect Dis. 2017 Apr 24;17:305. doi: 10.1186/s12879-017-2406-9 (PMC5404339; doi:10.1186/s12879-017-2406-9)
Supplement: Additional file 1: — Strategy and methods for the Treg measurements. (DOC 1009 kb) [file 12879_2017_2406_MOESM1_ESM.doc]

**Strategy and methods for the Treg measurements**

**Peripheral blood mononuclear cells isolation and culture**

Peripheral blood mononuclear cell (PBMC) isolation and cultures were carried out as previously described [1]. Briefly, PBMCs were first isolated through Ficoll-Paque gradient centrifugation and then 2.5 x10^6^ cells/well were cultivated for 96h in 24-well flat-bottomed plates with medium only, a cell wall preparation of *M. leprae* (MLCwA; 4 µg/mL), or, as positive control, the mitogen phytohemagglutinin, added for the last 48h of the culture (PHA; 5 μg/mL; Sigma), at 37°C and 5% CO_2_. The cells were then gently harvested from the wells, washed, and stained and analyzed for Treg frequency by flow cytometry as described below.

**Flow cytometry analysis of Tregs cells**

To assess the frequency of circulating Tregs and to analyze the expression of the suppressor molecule (CTLA-4) by these Tregs, ex-vivo PBMCs and cells harvested from the 96h PBMC cultures were incubated at 4˚C for 30 min with the following specific antibodies: anti-CD3 BV605 (clone SK7), anti-CD4 V500 (clone RPA-T4), anti-CD25 FITC (clone M-A251), anti-CD127 PE-Cy7 (clone HIL-7R-M21), anti-CD152 (CTLA-4) BV421 (clone BNI3) (all from BD Biosciences, San Diego, CA). After incubation, cells were washed with PBS/BSA. For the FoxP3 intracellular staining, cells were resuspended in Fix/Perm buffer (eBioscience, San Diego, CA, USA) and left at 4˚C for 30 minutes. Subsequently, these cells were washed with PBS/BSA and a permeabilization buffer (eBioscience), and incubated for 15 min with normal rat serum for preventing non-specific staining. Next, anti-FoxP3 antibody (clone PCH101, eBioscence) was added and the cells were incubated for 30 minutes at 4˚C. Cells were then washed and acquired and analyzed using LSRFortessa flow cytometer (BD Bioscience, San Jose, CA, USA). All analyses were performed in CD4+ cells gated within CD3+ cells. The gating strategies are shown in Figure 1. Data were processed using the FlowJo 7.6.5 software (Tree Star Inc., Ashland, OR, USA).

**
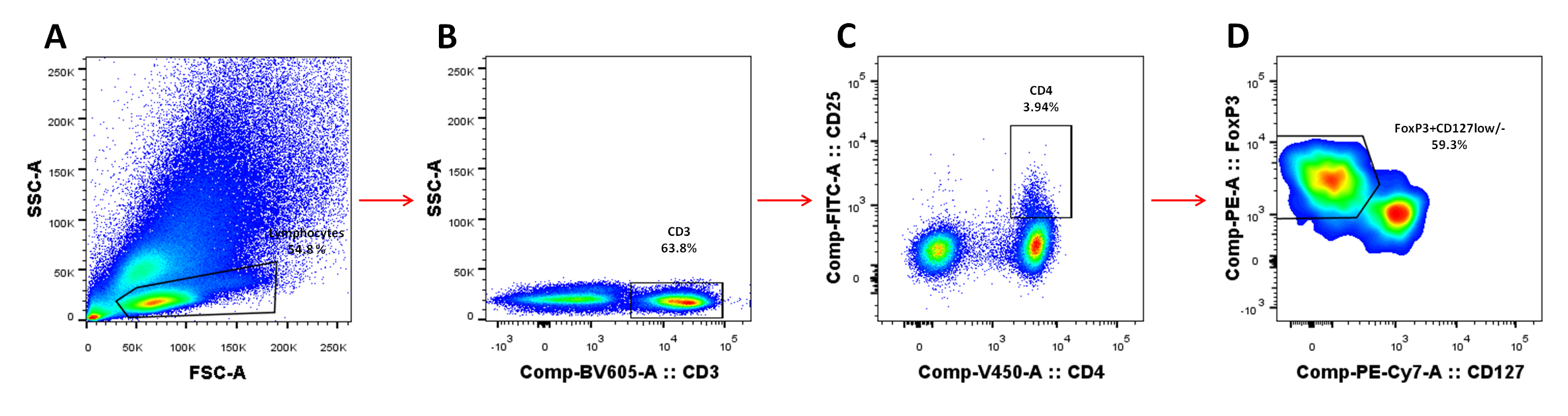
**

**Figure S1. Dot plots showing the gating strategy for characterization of the Treg population of the patient. A-C**, CD4/CD25 double-positive cells gated on the CD3^+^ lymphocyte population. **D**, gate on the FoxP3^+^CD127^low/-^ cells among the CD4^+^CD25^+^ cells.

**Immunohistochemistry staining for Foxp3 of the skin lesions biopsies**

Immunohistochemistry staining out as previously described [2]. Biopsies were taken with a standard dermatologic biopsy punch. Briefly, after deparafinization in xylene and hydration in ethyl alcohol, endogenous peroxydase was blocked by incubating in 3% hydrogen peroxydase solution in dark chamber. Antigen recovery was performed in a retrieval solution at pH 9.0 (S2368; Dako, Carpinteria, CA, USA) for 20 minutes at 95°C. Nonspecific proteins were blocked by incubating sections in skim milk. The primary antibody anti-Foxp3 (14-4776; eBioscience) as well as a labeled streptavidin–biotin complex (LSAB; Dako) were applied. 3,3-diaminobenzidine tetrahydroxychloride (DAB; Sigma) was used as chromogen, and the slides were counterstained with hematoxylin and hydrated in alcohol. The reaction was performed with positive and negative control. The latter comprised isotype control and omission of the primary antibody. The images were captured using AxioVision 4.8.2 software (Zeiss, Oberkochen, Germany). The area of the granulomatous inflammatory infiltrate was measured and the stained cells were counted using Image-Pro Plus, version 6.0 Media Cybernetics, Rockville, MD, USA).


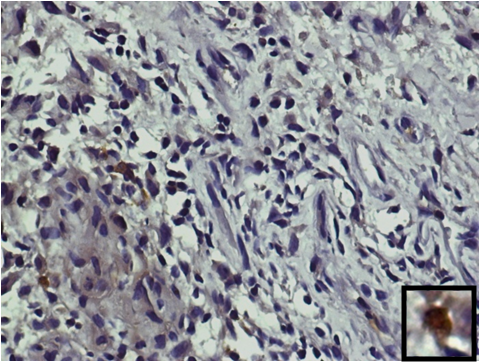


**Figure S2.** Representative section of immunofluorescence staining of FoxP3^+^ lymphocytes cells taken during upgrading T1R (x400).

References

# [**Vieira AP**](https://www.ncbi.nlm.nih.gov/pubmed/?term=Vieira AP%5BAuthor%5D&cauthor=true&cauthor_uid=26903606), [**Trindade MA**](https://www.ncbi.nlm.nih.gov/pubmed/?term=Trindade MÂ%5BAuthor%5D&cauthor=true&cauthor_uid=26903606), [**Pagliari C**](https://www.ncbi.nlm.nih.gov/pubmed/?term=Pagliari C%5BAuthor%5D&cauthor=true&cauthor_uid=26903606), [**Avancini J**](https://www.ncbi.nlm.nih.gov/pubmed/?term=Avancini J%5BAuthor%5D&cauthor=true&cauthor_uid=26903606), [**Sakai-Valente NY**](https://www.ncbi.nlm.nih.gov/pubmed/?term=Sakai-Valente NY%5BAuthor%5D&cauthor=true&cauthor_uid=26903606), [**Duarte AJ**](https://www.ncbi.nlm.nih.gov/pubmed/?term=Duarte AJ%5BAuthor%5D&cauthor=true&cauthor_uid=26903606), [**Benard G**](https://www.ncbi.nlm.nih.gov/pubmed/?term=Benard G%5BAuthor%5D&cauthor=true&cauthor_uid=26903606). Development of Type 2, But Not Type 1, Leprosy Reactions is Associated with a Severe Reduction of Circulating and In situ Regulatory T-Cells. [**Am J Trop Med Hyg.**](https://www.ncbi.nlm.nih.gov/pubmed/26903606)2016**;** 94(4):721-7.

1. Palermo ML, Pagliari C, Trindade MA, Yamashitafuji TM, Duarte AJS, Cacere CR, Benard G. [Increased Expression of Regulatory T Cells and Down-Regulatory Molecules in Lepromatous Leprosy](https://www.ncbi.nlm.nih.gov/pmc/articles/PMC3335697/). Am J Trop Med Hyg. 2012; 86(5): 878–883.
